# Clinical Profile and Treatment of Multiple Myeloma at a Tertiary Hospital in Kenya: A Five-Year Retrospective Review

**DOI:** 10.1155/2024/3208717

**Published:** 2024-02-12

**Authors:** Wanjiku Gichuru, Nicholas A. O. Abinya, Andrew Odhiambo, Fredrick C. F. Otieno, Simon Harrison, Matilda Ong'ondi

**Affiliations:** ^1^Department of Clinical Medicine and Therapeutics, University of Nairobi, Nairobi, Kenya; ^2^Nairobi Hospital Cancer Centre, Nairobi, Kenya; ^3^Peter MacCallum, Department of Oncology, Melbourne, VIC, Australia; ^4^Cancer Treatment Centre, Hemato-Oncology Unit, Kenyatta National Hospital, Nairobi, Kenya

## Abstract

**Background:**

Multiple myeloma (MM) is a chronic B-cell malignancy that involves proliferation of neoplastic clonal plasma cells in the bone marrow with circulating monoclonal immunoglobulins or constituent chains in serum or urine or both. It is a rare cancer with a lifetime risk of 0.76% and an age-adjusted incidence rate of 2.5–7.2 per 100,000 in high-income countries. There is a paucity of local data on the morbidity and treatment of MM.

**Methods:**

This was a single-centre descriptive retrospective study at the Kenyatta National Hospital (KNH). The study population included inpatients and outpatients with a documented diagnosis of MM managed between 1^st^ January 2014 and 31^st^ December 2018. Demographic data, pathology reports, laboratory results, and clinical findings were transcribed and uploaded to a database, and data analysis was done using Stata 16® software.

**Results:**

A total of 207 patient files were reviewed. The median age at presentation was 60 years with a slight male preponderance. Bone pain was the predominant complaint in 59% (139/207) of patients, with 17% of patients presenting with paraparesis or paraplegia. For patients who underwent imaging, osteolytic bone lesions were identified in 90.6% (126/139). Anaemia was present in 71% (147/207) patients, hypercalcemia in 55.4%, and renal dysfunction in 38.2%. There were 25 different treatment regimens prescribed, with 13 patients (7%) being on bortezomib-based triplet therapy.

**Conclusions:**

MM in KNH is a disease of the middle aged, affecting men and women almost equally and presenting mainly with bone pain and anaemia. Although there seems to be a general improvement in diagnosis and care, access to novel and less toxic agents for treatment is still wanting.

## 1. Introduction

Multiple myeloma (MM) is a chronic B-cell malignancy that involves proliferation of neoplastic clonal plasma cells in the bone marrow with subsequent overproduction of monoclonal immunoglobulins or its constituent polypeptide chains (paraproteins) in serum and urine, which lead to characteristic end-organ damage including renal dysfunction, anaemia, extensive lytic lesions, and hypercalcemia.

MM is a rare cancer, with a lifetime risk of 0.8% in the United States [1] and an age-adjusted incidence rate of 2.5–7.2 per 100,000 in Western countries [1, 2]. The reported incidence rate has been low in sub-Saharan Africa but is on the rise following improved diagnostic capabilities and increased life expectancy [3]. There are no specific population-level data available for MM in the Kenya cancer registry [4] and other East African registries [5]. However, there are a number of hospital-based studies that have looked at the incidence and clinical characteristics of MM in Kenya [6–8]. Following the updated diagnostic, pathological, and clinical criteria of MM internationally in 2014 and advances in new therapies including proteasome inhibitors and immunomodulators, there has been an increase in the overall survival of MM patients [9, 10].

This study was designed to document the clinical profile and treatment of multiple myeloma at the Kenyatta National Hospital to shed light on the current status of MM in Kenya.

## 2. Materials and Methods

This was a retrospective study conducted at the Kenyatta National Hospital (KNH), the largest referral hospital in Kenya with a bed capacity of 1800, receiving referrals from county and subcounty hospitals from around the country. KNH has dedicated outpatient oncology clinics, a radiotherapy cancer treatment centre (CTC), and inpatient oncology wards managing the bulk of cancer patients in Kenya. On average, a total of 500 to 600 cancer patients are seen every week. A digital filing system based on the ICD-10 classification allowed for retrieval of all file numbers corresponding to the ICD-10 C-90 diagnosis for MM in the hospital records.

Consent to use the patient records in the file was sought from the Kenyatta National Hospital Ethics Review Committee and was granted. Any identification data were removed, and patients were identified only by assigned study numbers.

The study population included patients with multiple myeloma classified under ICD-10 diagnosis C-90 (multiple myeloma excluding solitary plasmacytomas) managed at KNH between 1^st^ January 2014 and 31^st^ December 2018. All eligible cases were included. Data extraction was done, and study variables of interest were recorded into an online data extraction form and uploaded onto the Microsoft Excel database.

Categorical variables, e.g., sex, stage of disease, exposure to novel agent during treatment, and presence of supportive treatment modality, were reported as frequencies with percentages. Continuous data variables, e.g., age, were expressed as means and standard deviations if normally distributed or median and interquartile range if skewed. A multivariable Cox regression model was used to test the association between presence of anaemia, renal dysfunction, and hypercalcemia as independent variables and time to outcome (death). Censoring was done at date of the last-known follow-up for those in whom mortality was not witnessed. In building the model, stepwise backward and forward elimination at the 10% level of significance was used to select variables to be retained in the final model. The final model was fitted to determine the significant variables associated with mortality and reported as hazard rates. Significance was set at the 5% level of significance for a two-sided test. After model fitting, a test of the proportional hazards assumption was performed and it revealed no evidence of violation of this assumption.

The data were exported to the Microsoft Excel package and subsequently exported into a study STATA® file. Exploratory data analysis was done to identify missing values and check the skewness and normality of the data as well as check for significant associations.

## 3. Results

**Figure 1:**
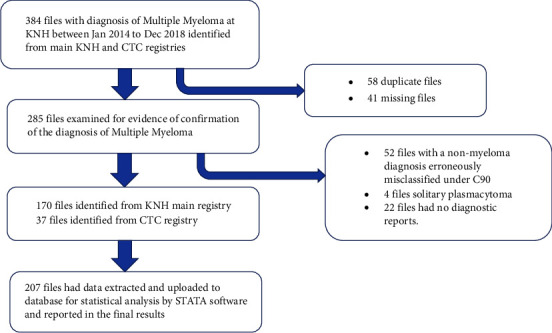
Flowchart of study recruitment.

A total of 384 file numbers were retrieved from the registries under the ICD classification of C-90 for multiple myeloma (Figure 1). There were 58 duplicate files (one patient with two file numbers) and 41 files missing from the registries. Four files of solitary plasmacytoma were excluded. In total, 207 files that met the study case definition of MM were retrieved and included in the final analysis.

### 3.1. Sociodemographic Characteristics

Patient baseline characteristics are presented in Table 1. The mean age was 58.5 years (SD 11.8), while the median age was 60 years (interquartile range, IQR: 50–66). The majority of patients (59%) were aged between 51 and 70 years, and only 6.5% were under 40 years of age. There was a slight male preponderance (*n* = 113, 54.6%) with a male to female ratio of 1.2 : 1.

### 3.2. Clinical Presentation

The prevalence of myeloma defining events including anaemia, renal dysfunction, hypercalcemia, and bone lysis was documented. In line with the IMWG diagnostic criteria, anaemia in this study was broadly defined as haemoglobin of less than 10 g/dl, which is determined as 2 g/dl (20 g/l) lower than the lower limit in the normal population of 12 g/dl as per the WHO criteria used in most epidemiological studies [11, 12]. Of the 147 patients who had anaemia, 88 (59.9%) had moderate anaemia (Table 2). Only 19 (8%) patients presented with symptoms documented by the clinician as suggestive of anaemia at diagnosis while 5 (2%) had bleeding tendencies.

Renal dysfunction was assessed based on laboratory findings, defined by the IMWG as serum creatinine >177 *μ*mol/l, and was found in 79/207 (38.2%) patients at diagnosis. Only 7 (3%) came in with overt fluid overload which may indicate renal failure. Other symptoms of renal dysfunction were not documented as a major presenting complaint at the point of diagnosis. Hypercalcemia was present in 127 (55.4%) of patients at diagnosis (*n* = 178).

Bone pain was the most common presenting complaint in 135 out of 207 patients (59%). Isolated lower back pain, without any other major complaint, was present in 76 (33%) patients, while lower back pain with paralysis and paresthesia of the lower limbs were recorded in 40 (17%) patients. Evidence of osteolytic bone lesions and/or compression fractures was seen in 126 patients (91.9%) out of 137 patients that had documented imaging. Magnetic resonance imaging (MRI) was the most common modality, done in 69 (50.4%) patients, 39 (28.5%) had a computer tomography (CT scan), and 29 (21.1%) had conventional radiography done.

### 3.3. Diagnostics and Staging

MM was diagnosed based on a bone marrow aspiration cytology report or tissue histology with one or more myeloma defining event (MDE). M-protein was detected in 114 (82%) of the 139 SPEP tests performed. The mean M-protein component at diagnosis was 35.52 g/l (SD 30.6 g/l) with a median of 33.8 g/l (IQR 5 -53 g/l). Other diagnostic tests included urine Bence-Jones protein testing, done in fifty-eight patients (25.3%), of which 42 (73%) were positive. Serum free light chain testing was performed in 16 (7.7%) patients. Ten patients (62.5%) had predominantly free kappa light chains, and six patients (37.5%) had predominantly free lambda light chains.

Only 9% had a serum beta-2 microglobulin report. Using the International Staging Score (ISS) for MM, 2 (10.51%) patients had stage 1 disease, 6 (31.5%) had stage 2, and 11 (57.9%) had stage 3 disease [13].

### 3.4. Treatment

Treatment was prescribed in 184 out of 207 patients (88.9%). For the other 23 patients (11.1%), the treatment prescribed was not on file; some patients died before treatment was prescribed, some discharged before treatment was initiated, while some had been referred for radiotherapy only, and their chemotherapy had not been documented.

The gold standard for MM treatment is induction therapy followed by autologous stem cell transplant (ASCT) for eligible patients. There were no patients who received ASCT in the study population.

There were 25 different combination treatment regimens for MM used within the study period (Table 3). Treatment regimens were varied; the majority were doublets and triplets based on medication available at the time (Table 3).

As supportive therapy, 73 patients (35.2%) required blood transfusions while 22 (10.6%) required dialysis. Bisphosphonates were prescribed in 82 patients (39.6%), most commonly zoledronic acid. Radiotherapy was performed in 83 patients (40%).

### 3.5. Outcome

There were 77 (33%) recorded deaths, from any cause, at date of the last-known follow-up on record. However, the outcome, dead or alive, in 130 patients (67%) at date of the last-known follow-up could not be ascertained from the records, and due to patient confidentiality, the patients or next of kin could not be contacted to provide information on outcome.

## 4. Discussion

Our study population was relatively young for MM with a median age of 60 years consistent with more recent studies in Kenya and Nigeria [8, 14]. An earlier study in Kenya by Othieno-Abinya et al. found a median age of 53 years [7]. The difference may be attributed to an increasing life expectancy in Kenya over the years, from 50 years in the year 2000 to 66.3 years in 2018 [15]. There was a slight male preponderance, consistent with regional haematological cancer data from Uganda and Nigeria [16, 17].

There was a heavy burden of MM-defining events (MDEs) in the study population which may be attributed to late presentation and delayed diagnosis. The presence of anaemia was high at 71%, similar to a study conducted in South Africa with an anaemia rate of 94%, also with a majority African population [18]. The normal range of the haemoglobin level in Kenya may actually be lower than that stated by WHO as documented in several studies [19, 20] and may contribute to the high prevalence of anaemia found in this study.

Over 90% of patients had evidence of osteolytic bone lesions and/or compression fractures on imaging at diagnosis. A large multinational systematic review conducted by Mohty et al. found the presence of lytic lesions in 67.5%–71.5% of patients [21], whereas this study had 91% of patients with multiple osteolytic lesions at diagnosis. Being a tertiary facility, most patients had prior imaging at presentation; hence, clinicians immediately ordered an MRI on the first review with the patients based on indication. The high uptake of MRI is attributed to the National Health Insurance Fund (NHIF) fully covering imaging costs for patients, making MRI more accessible.

A third of patients had high levels of plasma cell infiltration (>60%) which indicates severe disease as described by Kastritis et al. [22]. This would support that majority of patients presented in late-stage disease, likely ISS stage 3, as implied by the few who had a full staging work-up (9%). Resource-based adaptation of the international consensus staging guidelines for MM should be considered to better capture the epidemiology of the disease in LMIC with limited laboratory capabilities.

Despite the gold standard for MM treatment being induction therapy followed by autologous stem cell transplant (ASCT) for eligible patients [23, 24], no patients received ASCT in the study population. Currently, few African countries offer ASCT such as Algeria, Egypt, Morocco, Nigeria, South Africa, and Tunisia [25]. In 2023, one private facility in Nairobi, Kenya, embarked on offering ASCT; however, the cost is still prohibitive for most patients. The limited access to ASCT speaks to a large disparity in the quality of care offered to MM patients in Kenya and other sub-Saharan African countries as compared to high-income countries, where ASCT has led to a four-year overall survival rate of 81% compared to 65% and progression-free survival rate of 43 months compared to 22 months in patients receiving chemotherapy alone with melphalan, prednisone, and lenalidomide [26].

The 2019 Kenya national cancer treatment protocol recommends bortezomib-based regimens as first line for both transplant eligible and noneligible patients [27], in line with similar guidelines in Europe and North America [23, 24]. However, only 7% (13) of the patients at KNH were on triplet bortezomib-based regimen. The low uptake of bortezomib-based regimens may be related to the relatively prohibitive cost of bortezomib in the country, as it was not widely available and was not covered under the National Health Insurance Scheme then. The access is improving with coverage under the National Health Insurance Scheme; therefore, higher numbers are expected. Many different treatment combinations mirror the findings of a large prospective multinational noninterventional study on MM treatment carried out in Africa, Europe, and the Middle East by Mohty et al. that revealed great diversity in current treatment regimens used in MM [21]. They attributed this to the evolution of treatment regimens, as well as variable access to the increasing number of treatments. Clinician's prescriptions were likely to have been influenced by the availability of drugs locally and their affordability, as at times, patients had to pay out-of-pocket for what was not covered under the national scheme. The effect of this may be better explored in a prospective qualitative study looking at clinicians' decisions and the affordability and availability of MM medical treatments in our setting. This can be expanded to assess the real-world implementation of MM national treatment protocols and the impediments to their adaptation, a key aspect being their integration into what is covered under the National Health Insurance Scheme.

The study collected real-world data on myeloma management in a tertiary hospital in a low-resource setting. It was able to capture records of 207 patients over a period of five years, a relatively higher number than previous local studies. However, the study is not generalizable as it was a single-centre study. Another limitation was the reliance on obtaining data exclusively from the patient records.

## 5. Conclusion

Multiple myeloma at Kenyatta National Hospital is a disease of the middle aged, with a slight male preponderance and a high burden of disease at presentation. Staging of disease was inadequate with few patients able to do the full panel of tests. Treatment protocols were varied, and there is a need to improve the availability of newer agents so as to offer standardized protocols. A nationwide recommended standard-of-care protocol with basic minimum investigations would help in improving care and mobilizing policy-makers to facilitate in making these tests and medications accessible and affordable.

## Figures and Tables

**Table 1:** Sociodemographic characteristics of the MM patients at KNH.

Patient characteristics	Frequency	(%)
Total	*N* = 207	
Age mean	58.5 years	(SD 11.8)
Age median	60 years	(IQR 50–66)
Age categories (years)		
26–30	3	1.4
31–40	11	5.3
41–50	42	20.3
51–60	57	27.5
61–70	64	30.9
71–80	25	12.1
>80	5	2.4
Gender		
Male	113	54.6
Female	94	45.4
Employment status	[*n* = 121]	
Unskilled employment	63	52.1
Skilled employment	36	29.8
Retired	15	12.4
Unemployed	7	5.8

**Table 2:** Prevalence of myeloma defining events among multiple myeloma patients at KNH.

Myeloma defining events	Frequency	(%)
Anaemia	147/207	71
Mild (Hb 10 g/dl)	5 (3.4%)	
Moderate (Hb 7–9 g/dl)	88 (59.9%)	
Severe (Hb < 7 g/dl)	54 (36.7%)	
Renal dysfunction (creatinine levels >177 *μ*mol/l)	79/207	38.2
Hypercalcemia (calcium level >2.75 mmol/l)	127/178	55.5
One or more >5 mm osteolytic bone lesions	126/137	91.9

**Table 3:** Regimens used in the treatment of multiple myeloma at KNH.

Regimen	Frequency (%)
Chemotherapy-based	56 (30.4%)
Melphalan + prednisone	36
^*∗*^Other (melphalan + cyclophosphamide + prednisone cyclophosphamide + doxorubicin + vincristine etc)	20
Immunomodulator-based therapy	113 (61.4%)
Thalidomide-based	107 (94.7%)
Thalidomide + dexamethasone	45
Thalidomide + melphalan + prednisone	40
^*∗*^Other (cyclophosphamide + thalidomide + dexamethasone thalidomide + prednisone etc)	22
Lenalidomide-based	6 (5.3%)
Bortezomib-based therapy	15 (8.2%)
Bortezomib + thalidomide + dexamethasone	6
Bortezomib + lenalidomide + dexamethasone	6
Bortezomib + dexamethasone	2
Bortezomib + pomalidomide + dexamethasone	1

## Data Availability

The data supporting the study can be availed on request by e-mail to the corresponding author.

## References

[1] National Cancer Institute (2023). Surveillance, epidemiology and end results program. Multiple myeloma-cancer statistics. https://seer.cancer.gov/statfacts/html/mulmy.html.

[2] Kristinsson S. Y., Landgren O., Dickman P. W., Derolf ÅR., Björkholm M. (2007). Patterns of survival in multiple myeloma: a population-based study of patients diagnosed in Sweden from 1973 to 2003. *Journal of Clinical Oncology*.

[3] Cowan A. J., Allen C., Barac A. (2018). Global burden of multiple myeloma: a systematic analysis for the global burden of disease study 2016. *Journal of the American Medical Association Oncology*.

[4] Korir A., Okerosi N., Ronoh V., Mutuma G., Parkin M. (2015). Incidence of cancer in Nairobi, Kenya (2004–2008). *International Journal of Cancer*.

[5] AFCRN (2017). Kampala cancer registry report 2010-12. https://afcrn.org/images/Publication/Kampala-report-2010-12.pdf.

[6] Mukiibi J. M., Kyobe J. (1988). Pattern of multiple myeloma in Kenyans. *Tropical and Geographical Medicine*.

[7] Othieno-Abinya N. A., Abwao H. O., Nyabola L. O., Atinga J. A. O. (2005). Experience with multiple myeloma in a public referral hospital in Nairobi, Kenya. *Journal of Clinical Oncology*.

[8] Kiraka G., Etabale M., Riyat M. (2014). A review of 74 patients with newly diagnosed multiple myeloma at a tertiary referral hospital in Nairobi, Kenya. *African Journal of Cancer*.

[9] Durie B. G. M., Hoering A., Abidi M. H. (2017). bortezomib with lenalidomide and dexamethasone versus lenalidomide and dexamethasone alone in patients with newly diagnosed myeloma without intent for immediate autologous stem-cell transplant (SWOG S0777): a randomised, open-label, phase 3 trial. *The Lancet*.

[10] Attal M., Lauwers-Cances V., Hulin C. (2017). Lenalidomide, bortezomib, and dexamethasone with transplantation for myeloma. *New England Journal of Medicine*.

[11] Rajkumar S. V., Dimopoulos M. A., Palumbo A. (2014). International Myeloma Working Group updated criteria for the diagnosis of multiple myeloma. *The Lancet Oncology*.

[12] WHO (2011). *Haemoglobin Concentrations for the Diagnosis of Anaemia and Assessment of Severity*.

[13] Greipp P. R., Miguel J. S., Durie B. G. (2005). International staging system for multiple myeloma. *Journal of Clinical Oncology*.

[14] Nnonyelum O. N., Anazoeze M. J., Eunice N. O. (2015). Multiple myeloma in Nigeria: a multi-centre epidemiological and biomedical study. *The Pan African Medical Journal*.

[15] World Bank (2023). *Life Expectancy at Birth, Total (Years)-Kenya*.

[16] Wabinga H. R., Nambooze S., Amulen P. M., Okello C., Mbus L., Parkin D. M. (2014). Trends in the incidence of cancer in Kampala, Uganda 1991–2010. *International Journal of Cancer*.

[17] Akinbami A., Dada M. O., Dosunmu A. O., Balogun M. (2008). A adult haematooncology cases: a six year review at lagos state university teaching hospital ikeja. *The Internet Journal of Hematology*.

[18] Patel M. (2014). An epidemiological study of multiple myeloma in Southern Africa.

[19] Beutler E., West C. (2005). Hematologic differences between African-Americans and whites: the roles of iron deficiency and *α*-thalassemia on hemoglobin levels and mean corpuscular volume. *Blood*.

[20] Perry G. S., Byers T., Yip R., Margen S. (1992). Iron nutrition does not account for the hemoglobin differences between blacks and whites. *The Journal of Nutrition*.

[21] Mohty M., Terpos E., Mateos M. V. (2018). Multiple myeloma treatment in real-world clinical practice: results of a prospective, multinational, noninterventional study. *Clinical Lymphoma, Myeloma and Leukemia*.

[22] Kastritis E., Terpos E., Moulopoulos L. (2013). Extensive bone marrow infiltration and abnormal free light chain ratio identifies patients with asymptomatic myeloma at high risk for progression to symptomatic disease. *Leukemia*.

[23] Kumar S. K., Callander N. S., Alsina M. (2018). NCCN guidelines insights: multiple myeloma, version 3.2018. *Journal of the National Comprehensive Cancer Network*.

[24] Moreau P., San Miguel J., Sonneveld P. (2017). Multiple myeloma: ESMO Clinical Practice Guidelines for diagnosis, treatment and follow-up. *Annals of Oncology*.

[25] Harif M., Weisdorf D., Novitzky N. (2019). Special report: summary of the first meeting of african blood and marrow transplantation (AfBMT) group, casablanca, Morocco, april 19-21, 2018 held under the auspices of the worldwide network for blood and marrow transplantation (WBMT). *Hematology/Oncology and Stem Cell Therapy*.

[26] Palumbo A., Cavallo F., Gay F. (2014). Autologous transplantation and maintenance therapy in multiple myeloma. *New England Journal of Medicine*.

[27] Moh N. (2019). *National Cancer Treatment Protocols 2019*.

